# Compassion Satisfaction, Compassion Fatigue and Hardiness Among Nurses: A Comparison Before and During the COVID-19 Outbreak

**DOI:** 10.3389/fpsyg.2021.815180

**Published:** 2022-02-10

**Authors:** Mohammad Ali Zakeri, Elham Rahiminezhad, Farzaneh Salehi, Hamid Ganjeh, Mahlagha Dehghan

**Affiliations:** ^1^Non-communicable Diseases Research Center, Rafsanjan University of Medical Sciences, Rafsanjan, Iran; ^2^Social Determinants of Health Research Centre, Rafsanjan University of Medical Sciences, Rafsanjan, Iran; ^3^Student Research Committee, Razi Faculty of Nursing and Midwifery, Kerman University of Medical Sciences, Kerman, Iran; ^4^Nursing Research Center, Kerman University of Medical Sciences, Kerman, Iran; ^5^Clinical Research Center, Rafsanjan University of Medical Sciences, Rafsanjan, Iran; ^6^Department of Critical Care Nursing, Razi Faculty of Nursing and Midwifery, Kerman University of Medical Sciences, Kerman, Iran

**Keywords:** compassion satisfaction, compassion fatigue, hardiness, nurse, COVID-19

## Abstract

**Background:**

Nurses provide the majority of health-care services and face numerous health challenges during an epidemic. During the COVID-19 epidemic, nurses are subjected to physical, mental, and social disorders that impair their quality of life and hardiness. Therefore, it is important to be aware of the situation of nurses. The current study aimed to compare the compassion satisfaction, compassion fatigue and hardiness among nurses before and during the COVID-19 outbreak.

**Materials and Methods:**

This cross-sectional study included 508 clinical nurses from one public hospital in southern Iran. The subjects were recruited using census sampling methods in 2019–2020. Sampling was performed before (*n* = 266) and during the COVID-19 (*n* = 242) with a 1-year interval. Although, the study setting was the same before and during the COVID-19, questionnaires were completed by different nurses before and during the COVID-19. Demographic questionnaire, professional quality of life (ProQOL) questionnaire and Occupational Hardiness Questionnaire were used to collect data.

**Results:**

The scores of compassion satisfaction, compassion fatigue and hardiness did not differ significantly during the COVID-19 compared with before the COVID-19 (*p* > 0.05). Before COVID-19, hardiness and work experience predicted 11% of the variance of compassion satisfaction, whereas during COVID-19, hardiness and gender predicted 26% of the variance of compassion satisfaction. Before COVID-19, hardiness and work experience predicted 3% of the variance of compassion fatigue, whereas during COVID-19, hardiness, type of employment and gender predicted 6% of the variance of compassion fatigue.

**Conclusion:**

The current study found that compassion satisfaction, compassion fatigue and hardiness did not change during the COVID-19 outbreak compared with before the COVID-19 outbreak. However, during the COVID-19, the hardiness was a significant predictor of compassion satisfaction and compassion fatigue. The study results showed that it was possible to increase the compassion satisfaction and reduce the compassion fatigue by strengthening the hardiness of nurses. However, these results need to be considered in future studies, especially in crises such as COVID-19 disease.

## Introduction

COVID-19 first appeared in Wuhan, China in December 2019 and quickly spread throughout the world (Kisa, 2020). On March 11, 2020, the World Health Organization declared COVID-19 a pandemic (Sahin et al., 2020). Certainly, the general public has experienced anxiety, stress, fear, uncertainty, and insecurity as a result of the COVID-19 pandemic (Zakeri et al., 2021c,f). In this dire situation, nursing is one of the most important occupational groups, as well as one of the foundations of healthcare organizations. Due to its direct relationship with human health, the healthcare sector is now one of the most important areas of sustainable development in human societies (Zakeri et al., 2021b). Nurses provide the majority of the healthcare services in an epidemic (Wan et al., 2020), and they have the most contact with patients (Zakeri et al., 2021d). During the COVID-19 epidemic, front-line nurses face numerous health challenges (Wan et al., 2020) and are directly at risk when treating and caring for COVID-19 patients; as a result, severe stress and problems they experience at work lead to physical, mental, and social disorders (Kisa, 2020; Wan et al., 2020). Therefore, these factors can have an impact on nurses’ job performance and health, as well as their overall quality of life (Celmece and Menekay, 2020).

The concept of quality of professional life, which is related to personality traits and work environment of individuals (Zakeri et al., 2020) has two dimensions: compassion satisfaction and compassion fatigue (Lu et al., 2020; Zakeri et al., 2020). Compassion satisfaction refers to a person’s satisfaction with their ability to do their job well. Compassion satisfaction is one’s attitude toward their job that is associated with positive tendencies or feelings about one’s job (Zakeri et al., 2020). Compassion fatigue was firstly defined as an unpleasant psychological complication experienced by nurses and is divided into two parts: secondary traumatic stress and job burnout (Gerami Nejad et al., 2019). Employees with higher quality of professional life have a stronger organizational identity, higher job satisfaction, and are less likely to leave their jobs (Gerami Nejad et al., 2019; Zakeri et al., 2020). Before the COVID-19 outbreak, a meta-analysis study (2017) reported high prevalence of compassion satisfaction, compassion fatigue and burnout (Ruiz-Fernández et al., 2020a). Another study conducted on Australian emergency nurses also showed an average to high levels of compassion satisfaction and low to average levels of compassion fatigue (O’Callaghan et al., 2020). Celmece and Menekay (2020) found that stress, anxiety, and burnout had an impact on quality of life of healthcare workers caring for COVID-19 patients (Celmece and Menekay, 2020). Vafaei et al. (2020) reported a negative correlation between depression and quality of life among obstetric and gynecological healthcare providers during the COVID-19 outbreak. However, social support had a significant impact on improving quality of life (Vafaei et al., 2020). Nurses’ quality of life and job performance are influenced by their hardiness (Hatamipour et al., 2017).

Hardiness, a personality trait, serves as a source of resistance in the face of adversity. Hardy people have the ability to control life events, and they see problems as opportunities for advancement (Abdollahi et al., 2014; Teo et al., 2021). Maddi (2002) coined the term hardiness as a way to understand one’s relationship with others, goals, and problems. Hardiness consists of three parts: commitment, control, and challenge (Maddi, 2002; Saksvik-Lehouillier et al., 2016). Highly committed people believe in the importance, value, and meaning of who they are and what they do, and as a result, they can find meaning and arouse their curiosity in everything they do. People who have a strong sense of control see life events as predictable and controllable, and they believe that they can influence everything that happens around them. Individuals who are highly challenging believe that change is a natural part of life. These people see positive or negative situations as opportunities to learn and grow rather than as threats to their security and well-being (Maddi, 2002; Saksvik-Lehouillier et al., 2016; Teo et al., 2021). Vagni et al. (2020b) studied hardiness and coping strategies as mediators of stress and secondary trauma among emergency workers during the outbreak of COVID-19. They showed that hardiness and coping strategies helped reduce stress that predicted secondary trauma. Hardiness causes emergency personnel to be active and resistant, and to solve problems (Vagni et al., 2020b). Before the COVID-19 outbreak, Maramis and Cong (2019) studied the relationship between nurses’ hardiness personality and burnout. In this study, nurses had the highest level of hardiness (49.0%) and there was a poor but significant negative relationship between hardiness and burnout among nurses (Maramis and Cong, 2019).

A review of the literature comparing some variables before and after the COVID-19 pandemic indicates the need for further investigation into the impact of COVID-19 disease on these parameters. Zakeri et al. (2021e) showed that burnout did not change significantly during the COVID-19 pandemic compared with before the COVID-19. Zakeri et al. (2020) found that the mean scores of compassion satisfaction and burnout were 38.89 and 21.84, respectively, before the COVID-19 pandemic. However, Zhou et al. (2021) reported that these variables were 41.43 and 19.42, respectively, in the prevalence of COVID-19 disease. In addition, no study was found to compare compassion satisfaction, compassion fatigue and hardiness among nurses before and during the COVID-19 outbreak. Nurses are subjected to physical, mental, and social disorders during the COVID-19 epidemic (Zakeri et al., 2021a) that impair their quality of life and hardiness. Therefore, it is important to be aware of the situation of nurses. The current study aimed to compare the compassion satisfaction, compassion fatigue and hardiness among nurses before and during the COVID-19 outbreak.

## Materials and Methods

### Study Design and Setting

A cross-sectional study was conducted to investigate the compassion satisfaction, compassion fatigue, and hardiness of nurses in Rafsanjan before and during the COVID-19 outbreak.

### Sample Size and Sampling

In the present study, the study population consisted of 400 nurses before the COVID-19 disease and 500 nurses in the first wave of COVID-19 disease who worked in intensive care units, general wards and other medical wards. This study was performed in two times, before the COVID-19 and during the first wave of COVID-19 epidemic with 1 year apart. Ali Ibn Abi Talib Hospital was the only COVID-19 referral hospital in Rafsanjan city, Kerman province. Both groups of nurses were participated in the study by census sampling method. Inclusion criterion for nurses before the COVID-19 outbreak was at least 1 year of nursing experience and inclusion criteria for nurses during the COVID-19 were at least 1 year of nursing experience and at least 3 months of caring for COVID-19 patients. Nurses with mental disorders (based on self-report) and incomplete questionnaire were excluded from the study. Although, the study setting was similar before and during the COVID-19, questionnaires were completed by different nurses before and during the COVID-19.

Before the COVID-19 outbreak (from April to July 2019), 279 nurses completed the questionnaires (out of 400), with 13 of them being excluded from the study because of high missing value. Therefore, the effective response rate of nurses was 66.5% (*n* = 266). During the COVID-19 outbreak (from April to July 2020), 255 completed the questionnaires (out of 500), with 12 of them being excluded from the study because of high missing value, and one being excluded due to the history of mental disorders, so the effective response rate of nurses was 48.4% (*n* = 242). Finally, the data of 508 nurses were used in the final analysis.

### Measurements

A three-part questionnaire was used to collect data: socio-demographic characteristics, the professional quality of life questionnaire (ProQOL), and hardiness.

#### Socio-Demographic Characteristics

They include gender, age, marital status, educational level, income (million riyal), type of employment, work experience, ward, shift and working hours per month.

#### Professional Quality of Life Questionnaire

The ProQOL questionnaire consists of two sub-scales: (1) compassion satisfaction (10 items), (2) compassion fatigue (secondary traumatic stress and burnout include 20 items). Compassion fatigue is conceptualized through secondary traumatic stress and burnout. The questionnaire consists of 30 items that are scored on a five-point Likert scale (never = 1 to always = 5). The items (1, 4, 15, 17, and 29) are scored inversely (Stamm, 2010). High scores of compassion satisfaction indicates one’s satisfaction and ability to provide services and care whereas high scores of compassion fatigue reflect one’s vulnerability to disappointment and discomfort. Standard scores for domains are classified as high (above 42), moderate (41–23), low (below 22). Zakeri et al. (2020) translated and used the questionnaire in Iran. Instrument validity was determined by content validity, and its reliability was determined by Cronbach’s alpha coefficient. Reliability coefficients for CS, STS, and BO were 0.82, 0.8, and 0.47, respectively (Zakeri et al., 2020). In the present study, the Cronbach’s alpha coefficients for CS, STS, and BO were 0.86, 0.79, and 0.51, respectively.

#### Occupational Hardiness Questionnaire

Moreno-Jiménez et al. (2014) developed the Hardiness Questionnaire, which consists of 17 items on a 4-point Likert scale ranging from completely disagree (1) to completely agree (4) to assess occupational hardiness. It consists of three parts: control (3, 6, 9, 12, and 15), challenge (2, 5, 8, 11, 13, and 17), and commitment (1, 4, 7, 10, 14, and 16). People with a score higher than 45 are hardy while those with a lower score are not. Moreno-Jiménez et al. (2014) used the internal consistency method and Cronbach’s alpha coefficient to confirm the scientific validity and reliability of the Occupational Hardiness Questionnaire. Cronbach’s alpha coefficients were 0.76, 0.80, 0.74, and 0.86 for control, challenge, commitment, and the whole scale, respectively (Moreno-Jiménez et al., 2014). Akbari Balotanbegan et al. (2015) approved it among nurses in Iran using internal consistency method and Cronbach’s alpha coefficient. Cronbach’s alpha coefficient for the total scale was 0.88 (Akbari Balotanbegan et al., 2015). In the present study, the Cronbach’s alpha coefficients were 0.74, 0.73, 0.74, and 0.89 for control, challenge, commitment, and the whole scale, respectively.

### Data Collection

Participants were frontline nurses from two hospitals in Rafsanjan (*n* = 400). We collected data from nurses between April and July 2019 before the COVID-19 and from April to July 2020 during the COVID-19. Before and during the COVID-19, sampling was performed based on the socio-demographic characteristics, the professional quality of life (ProQOL) and hardiness. The researcher began sampling after obtaining the necessary permits and distributed questionnaires among eligible nurses. Nurses completed the questionnaires at an appropriate time in the presence of the researcher.

### Data Analysis

SPSS 25 was used for data analysis. Frequency, percentage, mean, and standard deviation were used to describe the participants’ characteristics, the ProQOL and hardiness levels. Independent *t* test was used to compare the ProQOL and hardiness levels before and during the COVID-19 outbreak. Independent *t* test, analysis of variance test and multivariate linear regression test were used to determine the correlates of ProQOL and hardiness before and during the COVID-19 outbreak. A significance level of 0.05 was considered.

### Ethical Considerations

The study protocol was approved by the Rafsanjan University of Medical Sciences (IR.RUMS.REC.1397.099 and IR.RUMS.REC.1399.135). At the beginning of the study and before the sampling, the researcher explained some information about the study objectives, confidentiality and anonymity of the information, and the voluntary participation. It was necessary to meet ethical standards, which meant that participation was voluntary and that all information was kept confidential. Participants were assured that their participation or withdrawal from the study would have no effect on their work and that all of their information would remain private. An informed written consent form was obtained from eligible nurses.

## Results

Table 1 shows the characteristics of the participants. The mean ages of the participants was 33.32 ± 6.12 and 33.07 ± 6.90 before the COVID-19 and during the COVID-19, respectively. The majority of the participants were female, married, had a bachelor’s degree, and had 3–5 years of work experiences.

**TABLE 1:** Comparison of the demographic characteristics of the participants before the COVID-19 and during the COVID-19.

Group Variables	Before COVID-19 (*n* = 266)				During COVID-19 (*n* = 242)			
	n (%)	Compassion satisfaction	Compassion fatigue	Hardiness	n (%)	Compassion satisfaction	Compassion fatigue	Hardiness
**Gender**								
Male	50 (18.8)	*t* = −0.77 (0.43)	*t* = 0.30 (0.75)	*t* = −0.18 (0.85)	68 (28.1)	*t* = −1.08 (0.28)	*t* = 1.34 (0.18)	*t* = 2.06 (0.04)
Female	216 (81.2)				174 (71.9)			
**Marital status**								
Unmarried/Widowed/Divorce	47 (17.7)	*t* = −1.30 (0.19)	*t* = −0.40 (0.68)	*t* = 2.46 (0.01)	59 (24.4)	*t* = −0.12 (0.90)	*t* = 0.44 (0.65)	*t* = −0.67 (0.50)
Married	219 (82.3)				183 (75.6)			
**Educational level**								
Bachelor	237 (89.1)	*t* = 0.41 (0.67)	*t* = −1.09 (0.27)	*t* = −0.22 (0.82)	223 (92.1)	*t* = 0.61 (0.54)	*t* = −1.02 (0.30)	*t* = 0.33 (0.73)
Master’s	29 (10.9)				19 (7.9)			
**Income (Million Riyal)**								
<3	106 (39.8)	*F* = 0.19 (0.82)	*F* = 0.89 (0.40)	*F* = 1.92 (0.14)	28 (11.6)	*F* = 0.78 (0.45)	*F* = 3.07 (0.04)	*F* = 1.11 (0.33)
3–5	141 (53.1)				177 (73.1)			
>5	19 (7.1)				36 (15.3)			
**Type of employment**								
Hired	164 (61.7)	*t* = 1.97 (0.04)	*t* = 0.56 (0.57)	*t* = −0.73 (0.42)	161 (66.5)	*t* = −0.46 (0.64)	*t* = 2.29 (0.02)	*t* = 0.33 (0.73)
Contract recruiters^a^/Committed^b^	102 (38.3)				81 (33.5)			
**Work experience (year)**								
>5	73 (27.4)				87 (36.0)			
5–10	120 (45.1)	*F* = 2.89 (0.03)	*F* = 1.95 (0.12)	*F* = 2.80 (0.04)	67 (27.7)	*F* = 0.83 (0.47)	*F* = 0.66 (0.57)	*F* = 0.95 (0.41)
11–15	40 (15.0)				38 (15.7)			
>15	33 (12.4)				50 (20.7)			
**Ward**								
Critical/intensive	76 (28.6)				89 (36.8)			
Emergency	44 (16.5)	*F* = 1.22 (0.94)	*F* = 0.35 (0.78)	*F* = 1.82 (0.14)	65 (26.9)	*F* = 2.09 (0.10)	*F* = 1.84 (0.14)	*F* = 1.57 (0.19)
Medical	90 (33.8)				59 (24.4)			
Others	56 (21.1)				29 (12.0)			
**Shift**								
Fixed	26 (9.8)	*t* = 0.98 (0.32)	*t* = −1.87 (0.06)	*t* = 0.72 (0.46)	23 (9.5)	*t* = 0.01 (0.98)	*t* = 0.22 (0.82)	*t* = −0.99 (0.32)
Rotational	240 (90.2)				219 (90.5)			
**Working hours (h) per month**								
<150	46 (17.3)				46 (19.0)			
150–160	100 (37.6)	*F* = 0.96 (0.40)	*F* = 0.83 (0.47)	*F* = 1.47 (0.22)	108 (44.6)	*F* = 1.23 (0.29)	*F* = 0.38 (0.76)	*F* = 4.14 (0.007)
161–170	80 (30.1)				49 (20.2)			
>170	40 (15.0)				39 (16.1)			

*Data were presented numerically (%). t, Independent t test; F, Analysis of variance test.*

*^a^Annually contracted with payment similar to hired nurses.*

*^b^It is obligatory to work for government for 2 years at a lower rate of pay.*

**FIGURE 1:**
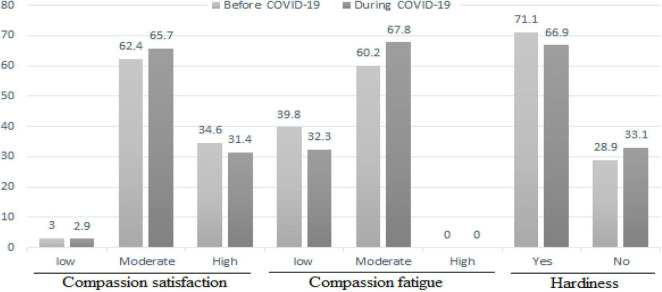
The comparison of the level of professional quality of life score and hardiness before the COVID-19 and during the COVID-19.

The mean scores of compassion satisfaction and compassion fatigue were 38.78 ± 6.57 and 47.87 ± 9.81, respectively, before the COVID-19. The mean scores of compassion satisfaction and compassion fatigue were 38.19 ± 6.52 and 49.70 ± 11.35, respectively, during the COVID-19. The compassion satisfaction and compassion fatigue scores did not change significantly during the COVID-19 compared with before the COVID-19 (*p* > 0.05) (Table 2). The majority of the participants had moderate level of compassion satisfaction and compassion fatigue before and during the COVID-19 (Figure 1).

**TABLE 2:** Comparison of the professional quality of life score among the participants before and during the COVID-19.

Group Variables	Before COVID-19 (*n* = 266)			During COVID-19 (*n* = 242)			Independent *t* test	Effect size	*P* value
	Median	Mean	SD	Median	Mean	SD			
Compassion satisfaction	39.50	38.78	6.57	38.00	38.19	6.52	1.02	0.13	0.31
Compassion fatigue	48.00	47.87	9.81	49.00	49.70	11.35	−1.94	0.17	0.06

*SD, Standard Deviation.*

The mean scores of hardiness were 46.92 ± 7.05 and 46.84 ± 7.14 before and during the COVID-19, respectively. The scores of hardiness and all its dimensions did not change significantly during the COVID-19 compared with before the COVID-19 (*p* > 0.05) (Table 3). The majority of the participants had hardiness before and during the COVID-19 (Figure 1).

**TABLE 3:** Comparison of the level of hardiness among the nurses before the COVID-19 and during the COVID-19.

Group Variables	Before COVID-19 (*n* = 266)			During COVID-19 (*n* = 242)			Independent *t* test	Effect size	*P* value
	Median	Mean	SD	Median	Mean	SD			
Control	15.00	14.31	2.28	15.00	14.20	2.52	0.49	0.05	0.62
Challenge	17.00	16.65	2.73	17.00	16.90	2.69	−1.05	0.09	0.29
Obligation	16.00	15.96	2.75	16.00	15.73	2.89	0.92	0.08	0.35
Hardiness	47.00	46.92	7.05	47.50	46.84	7.14	0.13	0.01	0.89

*SD, Standard Deviation.*

Multiple regression models were tested to see if study variables could predict compassion satisfaction, compassion fatigue, and hardiness before and during the COVID-19. Before the COVID-19, hardiness and work experience predicted 11% of the variance of compassion satisfaction, while hardiness and gender predicted 26% of the variance of compassion satisfaction during the COVID-19.

Before the COVID-19, hardiness and work experience predicted 3% of the variance of compassion fatigue, while, hardiness, type of employment, and gender predicted 6% of the variance of compassion fatigue during the COVID-19. Hardiness was found to be the best predictor of compassion satisfaction and compassion fatigue before and during the COVID-19 outbreak (Table 4). Marital status predicted 1% of the variance of hardiness before the COVID-19 (*R*^2^ = 1%). Working hours per month predicted 3% of the variance of hardiness during the COVID-19.

**TABLE 4:** The comparison of multiple regression analysis summary for compassion satisfaction, compassion fatigue and hardiness before COVID-19 and during COVID-19.

Variable	Multivariate regression									
	Before COVID-19 (*n* = 266)					During COVID-19 (*n* = 242)				
	Variable	*B*	95% confidence interval for B	*P* value	*R* ^2^	Variable	*B*	95% confidence interval for B	*P* value	*R* ^2^
Compassion satisfaction	Hardiness	0.16	0.04–0.26	0.007	11%	Hardiness	0.52	0.37–0.57	<0.001	26%
	Job history	0.12	0.02–1.66	0.043		Gender	0.13	0.41–3.59	0.014	
Compassion fatigue	Hardiness	−0.13	−0.35 to −0.02	0.026	3%	Hardiness	−0.19	−0.51 to −0.11	0.002	6%
	Job history	−0.17	−3.30 to −0.21	0.026		Type of employment	−0.17	−7.07 to −1.10	0.008	
	Type of employment	−0.13	−5.69 to 0.36	0.084		Gender	−0.13	−6.59 to −0.27	0.033	
Hardiness	Marital status	−0.15	−4.97 to −0.55	0.014	1%	Working hours per month	−0.15	−2.08 to −0.23	0.014	3%
	–	–	–	–		Gender	−0.12	−3.93 to 0.02	0.053	

*Data were presented as multiple regression analysis. Gender (male = 1 and female = 2); Job history (>5 = 1, 5–10 = 2, 11–15 = 3, and >15 = 4); Type of employment (Hired = 1 and Others Contract recruiters/Committed = 2); Working hours per month (<150 = 1, 150–160 = 2, 161–170 = 3, and >170 = 4); Marital status (Unmarried/Widowed/Divorce = 1 and married = 2).*

## Discussion

The current study aimed to compare compassion satisfaction, compassion fatigue, and hardiness among nurses before and during the COVID-19 outbreak. The results showed that the scores of compassion satisfaction and compassion fatigue did not change during the COVID-19 outbreak compared to before the COVID-19 outbreak. Most of the participants had relatively moderate levels of compassion satisfaction and compassion fatigue before and during the COVID-19 outbreak. Scores of hardness and its dimensions (commitment, control, and challenge) did not change significantly during the COVID-19 outbreak when compared to before the COVID-19 outbreak. Most of the participants were hardy both before and during the COVID-19 outbreak.

Trumello et al. (2020) showed that healthcare professionals dealing with patients with COVID-19 had lower levels of compassion satisfaction than those dealing with non-COVID-19 patients (Trumello et al., 2020). The reason for inconsistency was that in the present study, the level of compassion satisfaction was moderate and did not change during the COVID-19 outbreak compared to before the COVID-19 outbreak. Sampling was taken during two different times (before and during the epidemic) but the study of Trumello et al. (2020) was conducted during the epidemic. Zhou et al. (2021) showed that compassion satisfaction among healthcare professionals (nurse and physician) was moderate to high during the COVID-19 outbreak. Compassion satisfaction had a positive relationship with hand hygiene (Zhou et al., 2021) because compassion satisfaction was moderate during the COVID-19 outbreak, which is consistent with the present study. Dwyer et al. (2021) reported low levels of compassion fatigue and burnout, but moderate levels of compassion satisfaction among participants. Compassion satisfaction was notably higher than prior literature in the present study. The reason for inconsistency may be that in the present study, the scores of compassion satisfaction and compassion fatigue did not change during the COVID-19 outbreak compared to before the COVID-19 outbreak. On the other hand, the participants in the present study basically had high levels of compassion satisfaction (before the COVID-19 outbreak).

Ruiz-Fernández et al. (2020b) showed that healthcare professionals (physicians, nurses, etc.) had moderate to high levels of compassion fatigue and burnout during the COVID-19 epidemic in Spain. Physicians had higher scores of compassion fatigue and burnout, while nurses had higher compassion satisfaction scores (Ruiz-Fernández et al., 2020b). Ruiz-Fernández et al. (2020b) found that nurses’ scores of compassion satisfaction were moderate during the COVID-19 outbreak, which supported the present study. Ruiz-Fernández et al. (2020b) also showed that nurses’ scores of compassion fatigue were high during the COVID-19 outbreak, which did not support the present study. Maramis and Cong (2019) studied the relationship between nurses’ hardiness personality and burnout before the COVID-19 outbreak. In this study, nurses caring for inpatients had a high level of hardiness personality (Maramis and Cong, 2019).

Vagni et al. (2020a) showed that hardiness reduced stress, burnout and increased personal accomplishment among emergency workers during the COVID-19 outbreak. Park et al. (2018) showed that hardiness had an impact on the mental health of nurses during the MERS-CoV epidemic (Park et al., 2018). Park et al. (2018) found that the mean score of hardiness among nurses was lower than the mean score of hardiness among nurses in the present study. Hardiness, as shown in other studies (Bartone, 2006; Bartone et al., 2008), allows the individual to promote active attitudes, be committed to a goal, and perceive external situations, even negative ones, as opportunities to challenge. Hardiness can be a protective factor against the effects of stress and burnout (Vagni et al., 2020a). Hardiness, like other studies on health care workers involved during the COVID-19 outbreak, reduces the stress that predicts secondary trauma (Vagni et al., 2020b) and burnout (Vagni et al., 2020a). In the present study, hardiness was moderate during the COVID-19 outbreak and was an important predictor of compassion fatigue.

Frontline healthcare providers (physicians and nurses) not only experience mental health problems during an epidemic, but also their quality of professional life deteriorates long after the initial outbreak (Nathiya et al., 2021). It is important to monitor the mental health of health care providers, especially during this epidemic (Ortega-Galán et al., 2020). Compassion is an indicator of psychological well-being that includes cognitive and emotional functions with both voluntary and behavioral aspects (Zhang et al., 2018). Compassion satisfaction refers to the positive feelings and attitudes that people have toward their job (Zakeri et al., 2020). Compassion fatigue leads to avoidance or fear of dealing with certain patients, a decreased ability to empathize with patients or families, and mood swings (restlessness, irritability, hypersensitivity, anxiety, and anger), gastrointestinal, cardiovascular symptoms and sleep disorders (Zhang et al., 2018). In this specific situation, nurses could use their inherent motivation to care for patients as a means of gaining compassion satisfaction (Ruiz-Fernández et al., 2020b). The community’s genuine appreciation for the nurses’ efforts can strengthen the compassion of professionals who compromise their lives to help patients with COVID-19 (Alharbi et al., 2020). Continuing education programs (Zakeri and Dehghan, 2021) and compassion skills programs should be implemented to reduce compassion fatigue and improve compassion satisfaction and quality of life among professionals, as they can improve the patient quality of care and safety (Kim and Lee, 2020).

Hardiness, work experience, type of employment and gender were the variables that predicted compassion fatigue before and during the COVID-19. Zakeri et al. (2020) found that only clinical competence and job satisfaction were associated with compassion fatigue before the COVID-19. In Cetrano et al. (2017), study ergonomic problems and impact of work on life predicted higher levels of both compassion fatigue and burnout. Consistent with the results of the present study, Ruiz-Fernández et al. (2020b) showed that occupation and gender were associated with compassion fatigue during the COVID-19. We did not find further studies to compare these variables before and during the COVID-19 disease. Therefore, caution must be taken when interpreting the results. The outbreak of COVID-19 disease may have destructive effects on the condition of nurses. For the first time, the current study focused on the role of hardiness in compassion fatigue before and during the COVID-19. Hardiness is characterized by innate personality traits (Park et al., 2018). Gito et al. (2013) showed that hardy nurses had better mental health and hardiness was inversely related to stress. With good social support and proper stress management, healthcare workers in the COVID-19 unit should be more flexible (Nathiya et al., 2021) to achieve higher levels of hardiness in order to reduce compassion fatigue. However, managers should pay attention to the factors that affect hardiness and then try to increase the nurses’ hardiness using different modalities. Due to the small local sample and specific Iranian culture, it is necessary to consider these results in future studies and to conduct more detailed studies in larger groups and different cultures.

The main limitation of this study was that the data were obtained from a cross-sectional study, which did not allow for the examination of continuous variation of variables. Adaptation to conditions as well as measures currently being taken to adapt the workplace to new conditions (such as providing protective equipment or increasing the number of health care professionals, etc.) can affect these factors. Therefore, a follow-up study is essential over the next few months.

This study was performed on a small sample of local nurses in a center in southern Iran, so caution should be taken when interpreting the results. Comparison of these nurses with frontline nurses in other centers can be useful. Due to the lack of similar studies, it is necessary to be careful in interpreting the results and further studies on these variables is needed. Finally, the explained variance for compassion satisfaction and compassion fatigue is low (11 and 6%, respectively); therefore, more studies with a larger sample size are recommended to confirm the present study results.

## Conclusion

This study found that compassion satisfaction and compassion fatigue did not change among nurses during the COVID-19 outbreak compared to before the COVID-19. Scores of hardiness and its dimensions (commitment, control, and challenge) did not change significantly during the COVID-19 outbreak compared to before COVID-19. Due to the stressful situations among nurses who are in direct contact with COVID-19 patients, their mental health is important to provide health care in the COVID-19 epidemic. The study results show that we can be acquainted with the levels and changes in the variables of compassion satisfaction, compassion fatigue, and hardiness among nurses during the COVID-19 pandemic. Although, the present study did not show significant changes in these variables before and after the outbreak of COVID-19 pandemic, due to the lack of similar studies, further studies need to pay more attention to the factors affecting these variables. Policymakers need effective strategies to improve mental health and cope with critical situations as well as to increase the productivity of hospital staff. Efforts to strengthen nurses’ hardiness increase compassion satisfaction and reduce compassion fatigue. However, these results should be considered in future studies, especially in crises such as the COVID-19, which can have different effects on groups and communities, and future studies must address this crisis comprehensively and find ways to reduce these destructive effects. Therefore, the study of variables before and after pandemics can provide a different perspective toward selecting different interventions and strategies for improving compassion satisfaction, and hardiness and reducing compassion fatigue among nurses.

## Data Availability Statement

The raw data supporting the conclusions of this article will be made available by the authors, without undue reservation.

## Ethics Statement

The studies involving human participants were reviewed and approved by the Rafsanjan University of Medical Sciences. The patients/participants provided their written informed consent to participate in this study.

## Author Contributions

MZ and MD designed the study, provided critical feedback on the study and statistical analysis, and inputted to the draft of this manuscript. MZ, ER, FS, HG, and MD wrote the manuscript. HG collected the data. All authors have read and approved the final manuscript.

## Conflict of Interest

The authors declare that the research was conducted in the absence of any commercial or financial relationships that could be construed as a potential conflict of interest.

## Publisher’s Note

All claims expressed in this article are solely those of the authors and do not necessarily represent those of their affiliated organizations, or those of the publisher, the editors and the reviewers. Any product that may be evaluated in this article, or claim that may be made by its manufacturer, is not guaranteed or endorsed by the publisher.
